# Evaluating the Preliminary Outcomes and Acceptability of a Virtual Adaptation of Acceptance and Commitment Training for Caregivers of People With Disabilities (“I Am Not Alone”): Mixed Methods Study

**DOI:** 10.2196/86205

**Published:** 2026-03-25

**Authors:** Louisa L Y Man, Hadas Dahary, Soumya Mishra, Carly Magnacca, Lee Steel, Yona Lunsky, Alex Porthukaran, Jodie Siu, Nicole Bobbette, Brianne Redquest, Kendra Thomson, Melanie Penner, Jonathan Weiss, Johanna Lake, Kenneth Po Lun Fung

**Affiliations:** 1 Campbell Family Mental Health Research Institute Azrieli Adult Neurodevelopmental Centre Centre for Addiction and Mental Health Toronto, ON Canada; 2 Azrieli Adult Neurodevelopmental Centre Centre for Addiction and Mental Health Toronto, ON Canada; 3 School of Rehabilitation Therapy Queen's University Kingston, ON Canada; 4 Department of Applied Disability Studies Brock University St. Catharines, ON Canada; 5 Bloorview Research Institute Holland Bloorview Kids Rehabilitation Hospital Toronto, ON Canada; 6 Department of Psychology York University Toronto, ON Canada; 7 Adult Psychiatry and Health Systems University of Toronto Toronto, ON Canada; 8 Department of Psychiatry University of Toronto Toronto, ON Canada; 9 Toronto Western Hospital Toronto, ON Canada

**Keywords:** neurodivergent, family caregivers, clinicians, acceptance and commitment training, virtual adaptations

## Abstract

**Background:**

Family caregivers of children and youth with neurodevelopmental disabilities report higher levels of stress, anxiety, and depression than other caregivers, yet few evidence-based mental health services are available to support them. Our previous research demonstrated that caregivers benefitted from our in-person group-based acceptance and commitment training (ACT) workshop, which increased their psychological flexibility and improved their mental well-being. During the COVID-19 pandemic, we adapted this intervention to be delivered virtually.

**Objective:**

In this study, we evaluated the program’s impact on caregivers’ psychological flexibility and mental well-being, as well as the acceptability of the virtual program.

**Methods:**

A total of 205 family caregivers of neurodivergent people or people with disabilities who registered for a virtual, group-based ACT intervention (10-12 hours across 5 or 6 weeks) from 1 of 27 teams across Canada participated in this research. ACT process (measured via the Cognitive Fusion Questionnaire, Acceptance and Action Questionnaire, Self-Compassion Scale–Short Form, and Valued Living Questionnaire) and mental well-being outcomes (measured through the Patient Health Questionnaire, Parenting Stress Index, Multi-System Model of Resilience Inventory, and Short Warwick-Edinburgh Mental Well-being Scale) were measured before and after the intervention and again at an 8-week follow-up. An acceptability survey was completed after the intervention, including items on satisfaction with virtual aspects of the intervention.

**Results:**

There was high intervention acceptability related to the virtual format (at least >80% satisfaction) despite variability in individual preferences (preferred in-person format: 28/91, 31%; preferred virtual format: 37/91, 41%; no preference: 26/91, 29%). Significant improvements were found for most ACT processes (fusion: *F*_2,279_=63.96; psychological flexibility: *F*_2,264_=38.81; values: *F*_2,244_=8.32; self-compassion: *F*_2,170_=33.59; *P*<.001 in all cases) and mental well-being measures (parental stress: *F*_2,236_=20.27; well-being: *F*_2,137_=22.89; mood: *F*_2,15_=23.12; resilience: *F*_2,17_=32.83; *P*<.001 in all cases). Pairwise comparisons between preintervention and follow-up showed maintenance of ACT skills (*P*<.001 in all cases; η_p_^2^ ranged from 0.09 to 0.40) and well-being improvement (*P*<.001 in all cases; η_p_^2^ ranged from 0.19 to 0.37). Direct content analysis of participants’ qualitative survey responses revealed that cognitive defusion, mindfulness, and acceptance were the most helpful ACT processes. We received feedback for modifications to formatting, pacing, content, and duration of the group to support a wider range of caregiver needs.

**Conclusions:**

The virtual, group-based ACT intervention was acceptable and led to positive changes in psychological flexibility and mental well-being among family caregivers of people with disabilities or neurodivergent people, similar to outcomes observed with in-person delivery of the intervention. Further comparison and contrast with in-person programming is needed to ensure accessibility and sustainability of this program.

## Introduction

Family caregivers caring for children and youth with disabilities, including those with medical conditions (eg, epilepsy and diabetes) and/or neurodevelopmental conditions (eg, autism, intellectual disability, cerebral palsy, and fetal alcohol spectrum disorder), hereinafter referred to as caregivers of people with disabilities, experience both rewards and challenges. These caregivers are often balancing the needs of their family member with a disability, while attending to their own physical and mental well-being [1-5]. As a result, caregivers often face more stress and decreased well-being compared to caregivers of children without disabilities [6-8]. With heightened strain on the family system, caregivers may engage in avoidance strategies to cope [9]. While avoidance coping may offer short-term relief, it is linked to heightened stress and long-term challenges with mental well-being [10].

Interventions directly addressing caregiver well-being have been underprioritized, with most efforts focused on training caregivers to support their family member with a disability [11,12]. Among the limited research exploring caregiver-focused interventions, mindfulness- and acceptance-based approaches have received attention for enhancing caregivers’ mental well-being, with most studies focusing on adaptations of mindfulness-based stress reduction [13-15]. Another third-wave intervention, acceptance and commitment training (ACT), has also recently gained traction, highlighting the potential benefits of increasing psychological acceptance and value-driven behaviors, in addition to providing training on the principles of mindfulness. (For this manuscript, we used the term “acceptance and commitment training” rather than “therapy.” Our program focuses on providing ACT skills to a nonclinical population outside of a psychotherapeutic setting, so we chose to use “training” to align with previous studies of a similar nature [16].) Preliminary research exploring ACT, including multiple randomized controlled trials [17-20], has shown promising results in supporting the mental well-being of caregivers of people with disabilities [12,21-25].

ACT aims to increase psychological flexibility by opening up, being present, and doing what matters [21,26-28]. It acknowledges that life experiences evoke negative emotions and proposes that accepting and distancing from internal experiences can reduce long-term distress [27]. In offering an alternative way to engage with internal experiences, ACT can support caregivers to examine their values and commit to them through action, including prioritizing their well-being, often overlooked amid the pressures of caregiving.

Self-compassion is also a key factor in reducing caregiver stress. For caregivers of individuals with disabilities, greater self-compassion has been linked to lower levels of stress and depression as well as improved resilience [29]. A meta-analysis of 15 studies highlighted that self-compassion had moderate to large effects on parental stress, anxiety, and well-being [30]. ACT, with its focus on acceptance and values, has also been effective at improving self-compassion among mothers of children with neurodevelopmental disabilities [31] and people managing chronic pain [32]. Furthermore, in a randomized controlled trial of a single-session ACT workshop, psychological flexibility was found to be a mediator of self-compassion [33].

Consistent with the growing evidence demonstrating the benefits of caregiver-focused interventions led or supported by peer mentors [34-37], our model of ACT intervention is cofacilitated by teams of trained caregivers of people with disabilities and clinicians [22,38]. Caregivers’ insights contribute invaluable perspectives, while their presence fosters a supportive environment where participants feel validated, facilitating open dialogue and promoting mutual learning [16]. This collaborative approach enriches both the intervention experience and provides a sense of community among participants and facilitators.

The COVID-19 pandemic necessitated the transition from in-person interventions to virtual formats. Continuing to offer virtual support from that time has enabled health care providers to reach a broader audience of caregivers, including those who may prefer or require remote access to care due to a variety of reasons (eg, childcare responsibilities, geographical restrictions, and financial costs) [39].

Although in-person ACT has shown effectiveness for caregivers [22,39] and other virtual interventions are acceptable and effective [40], no research has examined the preliminary outcomes of a group-based virtual ACT for caregivers. Group-based virtual ACT has been found to be effective in addressing other concerns during the pandemic, including mental health for women [41], depression [42], and early psychosis [43]. This study aims to fill this gap by examining acceptability and preliminary outcomes of a virtual, group-based ACT intervention cofacilitated by caregivers and clinicians. Given previous evaluations of the main content, we hypothesized replication of good acceptability (with variations in accessibility due to virtual implementation), improvement in mental health outcomes, and increased ACT-related skills.

## Methods

### Overall Design

The study used a within-participants preintervention, postintervention, 8-week follow-up design to evaluate the acceptability and preliminary outcomes of a virtual ACT group intervention aimed at improving specific ACT processes and the mental well-being of caregivers of people with neurodevelopmental disabilities. Acceptability was examined through a Likert scale measuring intervention satisfaction and accessibility. Preliminary outcomes were evaluated through standardized measures of mental well-being and ACT-related process outcomes. Directed content analysis was used to examine participants’ postintervention responses to open-ended questions about program takeaways and suggestions for improving the intervention.

### Procedure

#### Participant Recruitment and Research Participation

Eligible participants included English-speaking caregivers of people with disabilities with internet access and a private space for the virtual intervention. Participants were recruited at 12 organizations serving people with disabilities and their families across Canada through digital flyers. Recruited participants of the ACT intervention were invited to take part in the research evaluation, which was not mandatory. ACT groups were run between 2020 and 2024. Consenting participants completed an online survey 1 to 2 weeks prior to the start of the intervention (preintervention time point), immediately after the intervention (postintervention time point), and again 8 weeks after the final session (follow-up time point). All study data were collected and managed using Research Electronic Data Capture (REDCap; Vanderbilt University) electronic data capture tools. Given the focus on implementation and delivery of services to partnering agencies, no previous sample size analysis was calculated; instead, the full dataset of individuals recruited during a grant period was analyzed.

#### Ethical Considerations

Participants were compensated with CAD $50 (US $37) for their time. This project received ethics approval from the Centre for Addiction and Mental Health Research (037-2017). Written informed consent was obtained from participants. Data were deidentified after data collection to safeguard participant information.

#### ACT Intervention Description

The ACT intervention consisted of 90-minute weekly virtual group sessions conducted via platforms (eg, Zoom or Webex) over 5 or 6 weeks (ie, modified longer version used by some organizations). The intervention was designed to accommodate groups of up to 15 participants, depending on site capacity. Each session was cofacilitated by at least 1 caregiver and 1 clinician, both of whom completed formal ACT facilitator training, including coaching sessions with experienced caregiver facilitators. The intervention was structured using a combination of didactic teaching, experiential activities, and small group sharing, with each session focused on learning about 1 or 2 specific ACT processes [22,38]. Facilitators implemented the intervention with high fidelity (84.7%) based on an internally designed fidelity measure where facilitators reported on whether they completed, partially completed, or did not complete specific exercises.

### Measures

#### Acceptability

Participants completed 7 items pertaining to their satisfaction with the intervention (eg, intervention relevancy, helpfulness, and usefulness). Seven items were measured on a 5-point Likert scale (1=strongly disagree and 5=strongly agree).

#### Well-Being Measures

##### Parenting Stress Index–Fourth Edition

The 6-item isolation and the 5-item health subscales of the Parenting Stress Index–Fourth Edition [44] were summed to reflect participants’ perceived social isolation and physical health in relation to parenting. Items are rated on a 5-point scale (1=“strongly agree” to 5=“strongly disagree”), with higher scores indicating more overall parental stress. The internal consistency was acceptable in this sample (Cronbach α=0.78 altogether; Cronbach α=0.64 for the Parenting Isolation subscale alone; Cronbach α=0.75 for the Parenting Health subscale alone).

##### The Short Warwick-Edinburgh Mental Well-Being Scale

The Short Warwick-Edinburgh Mental Well-being Scale **(**SWEMWBS) [45] is a 7-item measure designed to assess mental well-being. Items are rated on a 5-point scale (1=“none of the time” to 5=“all of the time”), with higher scores reflecting increased mental well-being. The internal consistency was good (Cronbach α=0.85).

##### Patient Health Questionnaire–4

The Patient Health Questionnaire–4 (PHQ-4) [46] is a valid ultrabrief measure for capturing anxiety and depressive symptoms. Total possible scores on the PHQ-4 can range from 0 to 12, with higher scores indicating greater challenges to mental well-being related to anxiety and/or depression. The internal consistency was good (Cronbach α=0.87).

##### Multi-System Model of Resilience Inventory

The Multi-System Model of Resilience Inventory (MSMR-I) [47] is a 27-item tool to measure resilience. The items examine 3 systems of resilience resources, including internal resources; external resources; and values, pursuits, and coping. Questions are scored on a 4-point Likert-type scale (0=“not at all like me” and 3=“very much like me”), with higher scores indicating greater resilience. The internal consistency was good (Cronbach α=0.81).

#### ACT Process Measures

##### Cognitive Fusion Questionnaire

The Cognitive Fusion Questionnaire (CFQ) [48] is a 7-item measure used to assess cognitive fusion vs defusion (ie, not relating to thoughts based on their literal content or having more psychological distance from one’s thoughts). Participants rate statements related to their struggle with their thoughts on a 7-point Likert scale (1=“never true” and 7=“always true”), with higher scores indicating greater fusion with thoughts and lower scores indicating greater defusion. The internal consistency was excellent (Cronbach α=0.92).

##### Acceptance and Action Questionnaire: Intellectual Disability Parent Version

The Acceptance and Action Questionnaire–Intellectual Disability Parent Version (AAQ-ID) [49] assesses psychological flexibility in relation to parenting a child with a disability. Participants rated 8 statements that were adapted to refer to a “child with a disability” (rather than “child with fetal alcohol syndrome” in its original form) using a 7-point scale (1=“never true” and 7=“always true”). Higher scores indicate less flexibility, while lower scores indicate more flexibility. The internal consistency was good (Cronbach α=0.83).

##### Valued Living Questionnaire

The Valued Living Questionnaire (VLQ) [50] is a two-part instrument used to assess (1) the perceived importance of values across 10 different areas of life (eg, work, family, and friends) on a 10-point scale and (2) the consistency of one’s actions with regard to their own values in each of these areas on the same scale. VLQ scores are calculated by the sum of the products of each pair of items (importance × consistency) across the 10 domains. Higher scores indicate greater alignment with values. The internal consistency was good (Cronbach α=0.82).

##### The Self-Compassion Scale–Short Form

The Self-Compassion Scale–Short Form (SCS-SF) [51] is a 12-item measure that assesses the ability to demonstrate care and kindness toward oneself and acceptance of one’s own imperfections. Responses are recorded using a 5-point Likert scale (1=“almost never” to 5=“almost always”), yielding a total score between 12 and 60, with higher scores indicating greater self-compassion. The internal consistency was good (Cronbach α=0.88).

### Data Analysis

#### Preintervention, Postintervention, and Follow-Up Quantitative Comparisons

A within-participants repeated measures ANOVA was conducted using SPSS (IBM Corp) to evaluate changes in mental well-being and ACT process outcomes (psychological flexibility, fusion, and values) across the 3 time points: preintervention, postintervention, and follow-up. Assumptions for repeated measures ANOVA were assessed by checking normality and the absence of outliers. Normality was evaluated using skewness, kurtosis z values, and *QQ* plots, while boxplots were examined visually for outliers. Violations of normality and outlier assumptions were noted for a couple mental well-being measures; specifically, 2 influential outliers were removed for the SWEMWBS and 3 for the PHQ-4. Moreover, sphericity was assessed using the Mauchly test, and when this assumption was violated, results were adjusted using the Greenhouse-Geisser correction. To account for multiple comparisons, Bonferroni correction was applied to post hoc contrasts, setting the significance threshold at *P*=.02 (ie, *P*=.05/3). When significant results were found, pairwise comparisons were performed for preintervention to postintervention, preintervention to follow-up, and postintervention to follow-up, with Bonferroni corrections applied. The analyses included data only from participants who completed the relevant measure at all 3 time points. Effect sizes were explored using partial eta squared (η_p_^2^) to assess the magnitude of the observed effects. To handle missing data, if 80% or more of the individual items were completed, the total score was calculated; otherwise, the data were considered missing. Only data with all 3 time points were included. The SCS-SF, SWEMWBS, PHQ-4, and MSMR-I were added in later cycles, resulting in smaller sample sizes for those analyses.

#### Content Analysis

Directed content analysis [52] was used to identify common concepts across three open-ended questions relating to participants’ experience with the intervention: (1) key intervention takeaways (n=96 completed responses), (2) suggestions for ways to improve the intervention (n=84 completed responses), and (3) most helpful ACT processes (n=70 completed responses). A mixture of deductive (ie, theory-driven codes about ACT processes [22] and self-compassion [53,54]) and inductive coding (ie, identified from the data) was used [52]. Open-ended responses were reviewed, and a codebook was codeveloped (LM and HD) and cocoded. Overarching category structure and interpretation were reviewed with a caregiver advocate (LS). After initial review, we found that the “key intervention takeaways” section had codes that were consistent with existing frameworks (eg, the self-compassion framework by Neff [53] and ACT processes), supporting the use of a directed content analysis approach [52]. Due to the brevity of other responses, we analyzed ways to improve the intervention and the most helpful ACT processes using summative content analysis.

## Results

### Demographics

**Figure 1:**
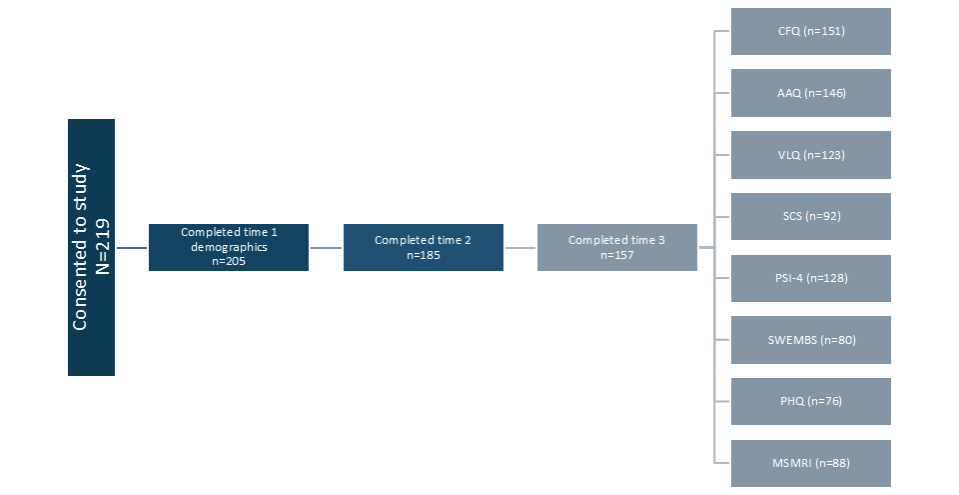
Participant flowchart showing the number of participants who consented and completed each of the 3 time points (preintervention, postintervention, and 8-week follow-up), as well as the number of complete datasets for each measure. AAQ-ID: Acceptance and Action Questionnaire–Intellectual Disability Parent Version; CFQ: Cognitive Fusion Questionnaire; MSMR-I: Multi-System Model of Resilience Inventory; PHQ-4: Patient Health Questionnaire–4; PSI-4: Parenting Stress Index–Fourth Edition; SCS-SF: Self-Compassion Scale–Short Form; SWEMWBS: Short Warwick-Edinburgh Mental Well-Being Scale; VLQ: Valued Living Questionnaire.

A total of 219 caregiver participants of people with disabilities from 27 virtual ACT intervention cohorts across Canada consented to participate in the research study between 2020 and 2024, of whom 205 (93.6%) proceeded to complete the demographics section. The participant flowchart is shown in Figure 1. Caregiver participants were aged between 19 and 78 (mean 47, SD 3) years. Approximately three-quarters of participants (170/205, 82.9%) identified as women, 12.2% (25/205) identified as men, 0.5% (1/205) identified as genderfluid, and 4.4% (9/205) did not report their gender. In total, 33.5% (70/209) of the participants obtained a postgraduate degree, 61.2% (128/209) attended or graduated from college or university, and 4.8% (10/209) attended high school equivalent or less (with 1 person declining to answer).

The age of participants’ family members with a disability ranged from 1 to 69 (mean 19, SD 1) years. The reported gender of 209 family members with disability indicated that 63.6% (n=133) were cisgender boys or men, 31.1% (n=65) were cisgender girls or women, 2.4% (n=5) were gender diverse (these included 1 each of transgender man, transgender woman, two-spirit, nonbinary, or gender neutral), and 2.9% (n=6) had missing data or preferred not to disclose. In terms of the 202 participants’ relationship with their family member with a disability, 59% (n=131) identified as biological parents; 14% (n=32) identified as adoptive parents; 13.4% (n=30) identified as siblings; and the remainder (n=9, 3%) identified as stepparents, grandparents, or step-grandparents. Table S1 in Multimedia Appendix 1 provides a breakdown of family members’ diagnoses.

### Quantitative Analyses

#### Acceptability of Virtual Delivery

Almost all participants reported that the web-conferencing software was easy to use (87/91, 95.6%), that they did not encounter technological issues (83/91, 91.2%), and that they were able to find a quiet environment to participate (85/90, 94.4%). Most participants (80/91, 87.9%) also felt connected in the virtual space. In terms of the format of the intervention, 30.7% (28/91) would have preferred an in-person intervention, 40.7% (37/91) preferred a virtual intervention, and 28.6% (26/91) did not have a preference for virtual or in-person intervention, highlighting that there were different preferences for both formats of this intervention.

#### Mental Well-Being

Table 1 presents the means, SDs, and the ANOVA results related to mental well-being variables at preintervention, postintervention, and follow-up. The ANOVA results revealed that time (ie, preintervention, postintervention, and follow-up) had a significant and large effect across measures of mental well-being.

**Table 1:** Repeated measures ANOVA comparing self-reported ratings of mental well-being at preintervention, postintervention, and 8-week follow-up.

	n	Preintervention, mean (SD)	Postintervention, mean (SD)	Follow-up, mean (SD)	*F* test (*df*)	*P* value	η_p_^2^
Parenting Stress Index–Fourth Edition	128	39.16 (8.22)	36.40 (8.49)	36.02 (8.07)	20.27 (2, 236.25)^a^	<. 001	0.14
Short Warwick-Edinburgh Mental Well-being Scale	80	19.35 (2.69)	20.84 (2.96)	21.36 (2.95)	22.89 (2, 137.72)^a^	<.001	0.23
Patient Health Questionnaire–4	76	5.13 (3.32)	3.59 (2.83)	3.12 (2.23)	23.12 (2, 15)	<.001	0.24
Multi-System Model of Resilience Inventory	88	41.50 (13.7)	47.52 (13.13)	49.86 (12.12)	32.83 (2, 17)	<.001	0.27

^a^Greenhouse-Geisser correction applied.

Paired comparisons with Bonferroni correction applied for the mental well-being outcome measures are detailed in Table 2. Contrasts revealed that scores on the Parenting Stress Index–Fourth Edition, PHQ-4, SWEMWBS, and MSMR-I significantly reduced from preintervention to postintervention and preintervention to follow-up, with no significant difference from postintervention to follow-up.

**Table 2:** Pairwise comparisons from preintervention to postintervention, postintervention to follow-up, and preintervention to follow-up assessments on mental well-being measures.

			*F* test (*df*)		*P* value		η_p_^2^
**Parenting Stress Index–Fourth Edition**							
	Preintervention to postintervention	23.08 (1, 127)		<.001		0.15	
	Postintervention to follow-up	0.70 (1, 127)		.41		0.01	
	Preintervention to follow-up	29.96 (1, 127)		<.001		0.19	
**Short Warwick-Edinburgh Mental Well-being Scale**							
	Preintervention to postintervention	19.23 (1, 79)		<.001		0.20	
	Postintervention to follow-up	4.60 (1, 79)		.04^a^		0.06	
	Preintervention to follow-up	36.38 (1, 79)		<.001		0.32	
**Patient Health Questionnaire–4**							
	Preintervention to postintervention	23.42 (1, 75)		<.001		0.24	
	Postintervention to follow-up	3.16 (1, 75)		.08		0.04	
	Preintervention to follow-up	35.16 (1, 75)		<.001		0.32	
**Multi-System Model of Resilience Inventory**							
	Preintervention to postintervention	34.31 (1, 87)		<.001		0.28	
	Postintervention to follow-up	5.50 (1, 87)		.02^a^		0.06	
	Preintervention to follow-up	51.85 (1, 87)		<.001		0.37	

^a^Not significant after adjusting for multiple comparisons.

#### ACT Processes

Table 3 presents the mean, SD, and ANOVA results related to the ACT process variables at preintervention, postintervention, and follow-up. Overall, time had a significant effect across measures of ACT processes, with large effect sizes (ie, η_p_^2^>0.14) for CFQ, AAQ-ID, and SCS-SF scores and a medium effect size (ie, η_p_^2^>0.06) for VLQ scores.

**Table 3:** Repeated measures ANOVA comparing self-reported ratings of acceptance and commitment training processes at preintervention, postintervention, and 8-week follow-up.

	n	Preintervention, mean (SD)	Postintervention, mean (SD)	Follow-up, mean (SD)	*F* test (*df*)	*P* value	η_p_^2^
Cognitive Fusion Questionnaire	151	29.11 (8.45)	24.87 (7.54)	22.46 (8.83)	63.96 (2, 279.40)^a^	<.001	0.30
Acceptance and Action Questionnaire–Internalized Dysfunction	146	27.25 (8.47)	23.27 (8.28)	22.36 (7.90)	38.81 (2, 264.70)^a^	<.001	0.21
Valued Living Questionnaire	123	476.56 (155.02)	516.74 (147.65)	519.76 (146.83)	8.32 (2, 244)	<.001	0.06
Self-Compassion Scale–Short Form	92	31.89 (8.45)	37.09 (8.13)	38.21 (8.13)	33.59 (2, 170.04)^a^	<.001	0.27

^a^Greenhouse-Geisser correction applied.

As shown in Table 4, paired comparisons with Bonferroni correction applied revealed a significant reduction in CFQ scores preintervention to postintervention, postintervention to follow-up, and preintervention to follow-up. For the AAQ-ID, VLQ, and SCS-SF, there was a significant reduction in scores from preintervention to postintervention and preintervention to follow-up, with no significant difference between postintervention and follow-up.

**Table 4:** Pairwise comparisons from preintervention to postintervention, postintervention to follow-up, and preintervention to follow-up for acceptance and commitment training process measures.

			*F* test (*df*)		*P* value		η_p_^2^
**Cognitive Fusion Questionnaire**							
	Preintervention to postintervention	51.77 (1, 150)		<.001		0.26	
	Postintervention to follow-up	21.14 (1, 150)		<.001		0.12	
	Preintervention to follow-up	100.23 (1, 150)		<.001		0.40	
**Acceptance and Action Questionnaire–Internalized Dysfunction**							
	Preintervention to postintervention	40.14 (1, 145)		<.001		0.22	
	Postintervention to follow-up	3.43 (1, 145)		.07		0.02	
	Preintervention to follow-up	58.35 (1, 145)		<.001		0.29	
**Valued Living Questionnaire**							
	Preintervention to postintervention	11.96 (1, 122)		<.001		0.09	
	Postintervention to follow-up	0.07 (1, 122)		.90		<0.01	
	Preintervention to follow-up	12.13 (1, 122)		<.001		0.09	
**Self-Compassion Scale–Short Form**							
	Preintervention to postintervention	32.98 (1, 91)		<.001		0.27	
	Postintervention to follow-up	2.46 (1, 91)		.12		0.03	
	Preintervention to follow-up	57.04 (1, 91)		<.001		0.30	

### Qualitative Analysis

Directed content analysis was used to identify (1) key takeaways from the intervention, (2) ways to improve the intervention, and (3) the ACT processes that were most helpful.

#### Key Takeaways From the Intervention

We applied two different frameworks to guide the organization of key takeaways (categories are italicized) from the intervention: (1) core ACT processes and (2) the self-compassion model by Neff [53].

Participants *described building psychological flexibility by integrating ACT processes*. Participants spoke about embodying *cognitive defusion*, often naming the leaves on the stream exercise as a core takeaway. This exercise involves visualizing each thought placed on a leaf floating down a stream, with the goal of visualizing a different relationship with thoughts (ie, defusion). Others described *acceptance*, primarily around making room for positive and negative feelings. Among *values*, participants detailed a sense of reestablishing a value for self-care and prioritizing their own needs, putting them into *committed action*. *Mindfulness* was also often described in the context of specific meditation exercises that were helpful. Finally, 1 respondent shared about how *perspective-taking* and *self-as-context* had been helpful. Altogether, this demonstrated that participants were learning and applying ACT principles in their daily life in a meaningful way.

We identified 3 categories based on the self-compassion framework by Neff [53], including *self-kindness*, *common humanity*, and *mindfulness* (ie, overlapping with ACT processes). *Common humanity*, often phrased as “I am not alone,” appeared often as the most commonly reported takeaway.

Finally, some respondents shared about *learning other skills* that were not specific to the ACT intervention but which may have emerged from debriefing discussions. Examples are provided in Multimedia Appendix 1.

#### Most Helpful ACT Processes

Finally, participants were asked which ACT processes were most helpful. *Cognitive defusion* was named most frequently, followed by mindfulness and acceptance (refer to Multimedia Appendix 1 for frequencies and examples).

#### Ways to Improve the Intervention

When asked what changes they would make to the intervention, most participants reported *no changes*; however, a few provided feedback on *formatting*, *pacing*, *content*, and *duration* (refer to Multimedia Appendix 1 for examples). Formatting, pacing, and content changes included more opportunities to connect with group members and more time to review concepts. Others preferred a hybrid or in-person format. Duration-related changes included having additional sessions, shorter sessions, and offering the intervention at different times.

## Discussion

### Principal Findings

The virtual ACT intervention was designed as a rapid response to pandemic-related restrictions and evaluated across agencies for acceptability and preliminary outcomes. This program had strong acceptability and preliminary outcomes, with improvements in well-being and ACT processes, as discussed below. We found significant improvements postintervention across ACT processes and well-being outcomes, similar to the original in-person program [12,22]. Postintervention improvements were maintained for all well-being measures (ie, parental stress, mental well-being, low mood, and resilience) and all ACT processes (ie, defusion, psychological flexibility, values-oriented living, and self-compassion). This study is the largest study to date to examine mental health, well-being, and ACT process outcomes for a virtual ACT program for caregivers [17,20,55]. Critically, we were able to demonstrate improvement in preliminary outcomes of this program for caregivers beyond mothers-only [12,22] or parents-only groups [17,20,55], supporting a subset of siblings who may take over the role of caretaking as parents age. While previous studies targeted caregivers of autistic children, adolescents, or young adults [17,20-22,29,55], our sample included caregivers of adults with diverse neurodevelopmental disorders (eg, intellectual disability and fetal alcohol spectrum disorder).

### Acceptability

For this virtual adaptation, there was high acceptability related to the virtual format (>80% satisfaction) despite variability in individual preferences. Although previous studies focused on ACT programs for caregivers collected acceptability measures indirectly through dropout rates [20,21] and Likert ratings [12,24], this is the first large-scale study to examine qualitative feedback in depth. Participants provided suggestions for improving the intervention that may also highlight their diverse needs to enhance acceptability. Examples included offering the program at different times, providing shorter or longer versions, allowing for more time for connection, and adding visual or material supports (eg, workbooks) to accommodate different learning styles.

The pandemic gave rise to a number of virtual and telepsychology programs, particularly for family caregivers of people with disabilities [56-58]. In addition to supporting families during pandemic restrictions, virtual programs have met needs that in-person programs were not able to meet (eg, childcare, travel, and geographic restrictions) [56,58]. Importantly, when family caregivers in this study were polled after completing the intervention around future preferences for programming modality, the proportion of family caregivers who wanted the virtual or in-person format or had no preference was roughly the same (in-person format: 28/91, 31%; virtual format: 37/91, 41%; no preference: 26/91, 29%). This highlighted that both in-person and virtual adaptations of this program may exist to serve different needs. Offering both in-person and virtual adaptations may help address barriers relating to time, income, and childcare support [11,59,60]. Consequently, the virtual adaptation of the ACT program may broaden opportunities for people who are unable to attend in-person programs, with potential suggestions for adaptations to continue evolving to meet more diverse needs. However, considerations regarding how virtual programs may be less accessible to people without technology or a stable internet connection (eg, rural or remote communities) should be considered.

The current synchronous virtual adaptation may have relative advantages over asynchronous adaptations. There have been few studies on virtual asynchronous studies for family caregivers of people with neurodevelopmental disabilities. One meta-analysis of asynchronous (eg, websites, apps, and computer-based interventions) programs focused on parent-mediated interventions for autistic children demonstrated that asynchronous interventions may not produce any changes in outcome [61]. This could suggest that virtual asynchronous modalities alone may not be as effective as virtual synchronous modalities, but this possibility requires further evaluation. This virtual synchronous program may provide family caregivers with a sense of building connection and fostering common humanity [53] that is similar to the in-person format without the cost and barriers associated with an in-person group. Hybrid psychoeducational programs, such as the Healthy Mothers Healthy Families program, which involves asynchronous modules and synchronous training on physical health needs, were found to foster a sense of not being alone while providing mothers with time flexibility in their daily routine [56]. Altogether, this virtual adaptation of ACT for caregivers supported participants in feeling connected and empowered to follow their values, providing a possible relative advantage to asynchronous virtual modalities by building a real-time shared connection.

### Well-Being

We found significant improvements in postintervention well-being outcomes, consistent with previous in-person workshops [12,22]. Parental stress and depression symptoms were both reduced by the end of the program and maintained. Importantly, well-being and resilience also improved and were maintained. This suggests that a virtual adaptation of this program may help address the service gap for caregivers who experience higher levels of stress compared with those caring for children without disabilities [6-8]. This augments an array of existing programs that show reductions in caregiver stress [12,22,24] and improvements in mental well-being [17,20,21]. Notably, this is the first virtual adaptation of ACT for caregivers that has examined well-being outcomes, such as overall mental well-being and resilience factors, highlighting that virtual modality can be helpful for improving well-being for caregivers in need.

### ACT Processes

Finally, this caregiver study is the largest to date that examines ACT processes commonly examined, such as cognitive flexibility [20,21] and defusion [22], while also including additional processes such as self-compassion and values. We found significant improvements postintervention in all 4 ACT processes measured, including a reduction in cognitive fusion and psychological inflexibility and improvements in values-oriented living and self-compassion. Not only were the results maintained, but cognitive defusion continued to improve at the 8-week follow-up relative to the end of the program. This result may suggest that caregivers were continuing to deepen their practice of this skill beyond the program. This suggests that improvements in well-being may be related to the adoption of core ACT skills. When participants were asked about their core takeaways and the most helpful ACT processes from the group, participants regularly named cognitive defusion and provided examples (eg, list of cognitive defusion techniques). This provided further context for our finding that participants continued to increase their cognitive defusion skills by the 8-week follow-up, perhaps due to the concreteness of using this skill.

Participants additionally provided qualitative takeaways that matched the model of self-compassion by Neff [53], highlighting areas of self-kindness, common humanity, and mindfulness [54]. In particular, common humanity in the form of “I am not alone” was the most commonly provided answer as key takeaways from the program. This may be related to the cofacilitated model of our program, where caregivers with lived experience share their own personal examples and barriers, shifting the discussion from clinical professionals providing advice to more tangible, real-world examples. Altogether, participants demonstrated improvement across all ACT core processes and provided positive qualitative feedback on how these ACT processes continue to affect their lives after completion of the program.

### Limitations and Future Directions

Without a control group, it is not known whether changes reported here are instead attributable to the passage of time, although a previous randomized control trial of the in-person version of this program did suggest treatment effects [20]. Moreover, only 209 people consented to participate in the study (76-151 completing all measures), which may not represent those who could not access a virtual program (eg, due to internet or technology accessibility), who dropped out, or who did not consent to research. Future replication and comparison across modalities are needed to understand accessibility of virtual vs in-person programs.

Another limitation may be the differential impacts of caregivers with different training experiences, baseline ACT-related characteristics, and disability status. Future research is underway to examine how baseline fusion, psychological flexibility, caregiver experience, and the disability status of the person they care for influence outcomes of ACT.

### Conclusions

The virtual adaptation of ACT for family caregivers was found to be acceptable and fostered a sense of shared humanity, resulting in improved well-being and greater practice of defusion, psychological flexibility, and values alignment. This builds on the literature that highlights the rising need for virtual, synchronous programs that address family caregiver well-being, as well as the continued need for program modifications to address diverse needs.

## Data Availability

The datasets generated or analyzed during this study are not publicly available due to research participants not consenting to data sharing at the time of data collection but are available from the corresponding author on reasonable request.

## Appendix

Multimedia Appendix 1 Supplementary tables describing frequencies and percentages of family members’ diagnoses, participant quotes on key takeaways, participant quotes on suggested changes, and participant quotes on the most helpful acceptance and commitment training processes.

## Abbreviations

AAQ-ID: Acceptance and Action Questionnaire–Intellectual Disability Parent Version
ACT: acceptance and commitment training
CFQ: Cognitive Fusion Questionnaire
MSMR-I: Multi-System Model of Resilience Inventory
PHQ-4: Patient Health Questionnaire–4
REDCap: Research Electronic Data Capture
SCS-SF: Self-Compassion Scale–Short Form
VLQ: Valued Living Questionnaire
