# Supplementary material for: Evaluating the Preliminary Outcomes and Acceptability of a Virtual Adaptation of Acceptance and Commitment Training for Caregivers of People With Disabilities (“I Am Not Alone”): Mixed Methods Study
Source: JMIR Form Res. 2026 Mar 25;10:e86205. doi: 10.2196/86205 (PMC13062744; doi:10.2196/86205)
Supplement: Multimedia Appendix 1 [file formative_v10i1e86205_app1.docx]

**TITLE: “I am not alone”: Evaluating the Effectiveness and Acceptability of a Virtual Adaptation of Acceptance and Commitment Training for Caregivers of People with Disabilities**

**Supplementary Materials**

**Authors**

Man, L. L. Y.^1^, Dahary, H.^1^, Mishra, S.^1^, Magnacca, C. A.^1, v^, Steel, L.^1^,, Lunsky, Y.^1,2,^ , Porthukaran, A.^1^, Siu, J.^1^, Bobbette, N.^3^, Redquest, B. ^1^, Thomson, K.^1,9^, Penner, M.^8,9^, Weiss, J.^5^, *Lake, J^1,2,^ *Fung, K. P-L. ^2,4^.

^1^Azrieli Adult Neurodevelopmental Centre, Centre for Addiction and Mental Health

^2^Department of Psychiatry, University of Toronto

^3^Queen’s University

^4^Toronto Western Hospital

^5^York University

^6^University of Calgary

^7^Bloorview Research Institute, Holland Bloorview Kids Rehabilitation Hospital

^8^Department of Paediatrics, University of Toronto

^9^Department of Applied Disability Studies, Brock University

*joint senior authorship

**Corresponding Author**

Louisa Man
Azrieli Adult Neurodevelopmental Centre and Campbell Family Health Research
Centre for Addiction and Mental Health
[louisa.man@camh.ca](mailto:louisa.man@camh.ca)

**Supplementary Table 1.**

Frequency and percentage of family members’ diagnosis (i.e., *n* = 209 responses).

| **Diagnosis** | **Number** | **Percentage*** |
| --- | --- | --- |
| **Neurodevelopmental Disability** |  |  |
| Autism | 123 | 59 |
| Attention-Deficit/Hyperactivity Disorder | 82 | 39 |
| Intellectual Disability | 43 | 21 |
| Fetal Alcohol Spectrum Disorder | 32 | 15 |
| Learning Disability | 13 | 6 |
| Other Neurodevelopmental Disorders | 4 | 2 |
| **Mental Health/Psychological Concerns** |  |  |
| Anxiety | 62 | 30 |
| Obsessive Compulsive Disorder | 18 | 9 |
| Depression | 18 | 9 |
| Oppositional Defiant Disorder | 14 | 7 |
| Speech/communication difficulties | 8 | 4 |
| Other Mood Disorders | 7 | 3 |
| Attachment Disorder | 7 | 3 |
| Post-Traumatic Disorder | 4 | 2 |
| Personality Disorders | 4 | 2 |
| Behavioral Complexities Other | 2 | 1 |
| Eating Disorder | 2 | 1 |
| **Physical Concerns** |  |  |
| Physical Complexity | 46 | 22 |
| Genetic Complexity | 23 | 11 |
| Epilepsy | 22 | 11 |
| Physical Neural Complexities | 14 | 7 |
| Sensory Processing Disorder | 9 | 4 |
| Sleep Disorders | 7 | 3 |
| Cerebral Palsy | 6 | 3 |
| Hearing/Vision Loss | 6 | 3 |
| Motor Coordination Disorders | 4 | 2 |

*** - As some participants identified as having more than one family member with a disability and/or a family member with multiple diagnoses, the sum total is greater than 100%.

**Supplementary Table 2.**

Participant responses for each category identified regarding key takeaways, analyzed using Directed Content Analysis.

| Theme | Quote |
| --- | --- |
| ACT Process: psychological flexibility | “...the value and process involved psychological flexibility and how it can help myself and my family” |
| ACT Process: cognitive defusion | “distancing self from thoughts”  “letting worries go” |
| ACT Process: acceptance | “make space for all of my feelings (bad and good)”  “making room for acknowledging our feelings” |
| ACT Process: perspective-taking/ self-as-context | “stepping back and looking at situations from another point of view” |
| ACT Process: mindfulness | “staying present and being mindful in everyday activities (not borrowing worries)” |
| ACT Process: values | “we have to listen to ourselves and not just work beyond issues (ignore) as it took tolls some weeks on caregivers”  “I can connect to my values for purposeful actions” |
| ACT Process: committed action | “I was able to become focused on my self-care goal and put it into action” |
| Self-compassion: Self-kindness | “Don’t be so hard on myself”  “Be more compassionate and understanding of myself” |
| Self-compassion: common humanity | “I am not alone” |
| Learned other skills | “I can reframe my thinking whenever I want to (or I prompt myself to)”  “Being able to balance my emotions” |

Note: The question posed to participants is “Tell us about 3 things that you took away from this workshop.” The quotes above include full quotes provided by participants, which may appear disembodied and incomplete, but reflect participants actual brevity in quotes. This may be due to the fact that caregivers have limited time to write long answers.

**Supplementary Table 3**.

Participant open-ended responses to suggested changes, analyzed using Summative Content Analysis.

| Theme | Quote |
| --- | --- |
| **No change** | “everything was well run and engaged”  “no, it went so very well” |
| **Formatting, pacing or content changes** | “more chances to engage with other caretakers, we plan to keep in touch since classtime”  “I felt like we moved through things too quickly and that there needed to be more review/practice before introducing new techniques at each session”  “one weekend in person” |
| **Duration-related changes** | “probably could have had 2 additional sessions to fully incorporate some of the techniques”  “shorter duration of each workshop, maybe 1.5 hours max”  “the time: (redacted timeslot) was very difficult for me” |

Note: The question posed to participants is “Is there anything you would like us to change fo future workshops”? The quotes above include full quotes provided by participants, which may appear disembodied and incomplete, but reflect participants actual brevity in quotes. This may be due to the fact that caregivers have limited time to write long answers.

**Supplementary Table 4**.

Frequency and examples of most helpful ACT processes named by participants

| ACT Process | Frequency | Examples |
| --- | --- | --- |
| Cognitive Defusion | 32 | “Defusion is what I’ve been using mostly, being able to name what I’m feeling and put some distance” |
| Mindfulness | 30 | “Walking away - noticing things around me, being in the moment of nothing.” |
| Acceptance | 28 | “I think acceptance is the thing I have done best. Even though it is the hardest one.” |
| Values | 11 | “Recentering thoughts and choices to align with values”  “Values helped me realize how far my actions I drifted away from my beliefs while being immersed in caregiving” |
| Committed Action | 10 | “I also do my best to act in a way that’s aligned with my values.”  “Yes - restarting volunteering” |
| Barriers to accessing specific ACT processes | 5 | “I’m trying to remember the exercises, but I can’t. I wish there was a booklet summarizing each of the exercises.” |
| All | 3 | “They are all helpful” |
| Self-as-context | 0 | N/A |

*Note:* The question posed to participants is: Are there particular ACT processes (e.g., acceptance, defusion, contact with the present moment, self as context, values, committed action) you have found particularly helpful?
